# Low-Intensity Focused Ultrasound Technique in Glioblastoma Multiforme Treatment

**DOI:** 10.3389/fonc.2022.903059

**Published:** 2022-05-19

**Authors:** Rajneesh Mungur, Jiesheng Zheng, Ben Wang, Xinhua Chen, Renya Zhan, Ying Tong

**Affiliations:** ^1^ Department of Neurosurgery of the First Affiliated Hospital, School of Medicine, Zhejiang University, Hangzhou, China; ^2^ Key Laboratory of Cancer Prevention and Intervention, Key Laboratory of Molecular Biology in Medical Sciences, National Ministry of Education, Cancer Institute, The Second Affiliated Hospital, School of Medicine, Zhejiang University, Hangzhou, China; ^3^ Institute of Translational Medicine, Zhejiang University, Hangzhou, China; ^4^ Key Laboratory of Pulsed Power Translational Medicine of Zhejiang Province, Hangzhou, China; ^5^ Department of Hepatobiliary and Pancreatic Surgery of the First Affiliated Hospital, School of Medicine, Zhejiang University, Hangzhou, China

**Keywords:** glioblastoma, low-intensity focused ultrasound, drug-delivery, blood-brain barrier, blood-tumor barrier (BTB)

## Abstract

Glioblastoma is one of the central nervous system most aggressive and lethal cancers with poor overall survival rate. Systemic treatment of glioblastoma remains the most challenging aspect due to the low permeability of the blood-brain barrier (BBB) and blood-tumor barrier (BTB), limiting therapeutics extravasation mainly in the core tumor as well as in its surrounding invading areas. It is now possible to overcome these barriers by using low-intensity focused ultrasound (LIFU) together with intravenously administered oscillating microbubbles (MBs). LIFU is a non-invasive technique using converging ultrasound waves which can alter the permeability of BBB/BTB to drug delivery in a specific brain/tumor region. This emerging technique has proven to be both safe and repeatable without causing injury to the brain parenchyma including neurons and other structures. Furthermore, LIFU is also approved by the FDA to treat essential tremors and Parkinson’s disease. It is currently under clinical trial in patients suffering from glioblastoma as a drug delivery strategy and liquid biopsy for glioblastoma biomarkers. The use of LIFU+MBs is a step-up in the world of drug delivery, where onco-therapeutics of different molecular sizes and weights can be delivered directly into the brain/tumor parenchyma. Initially, several potent drugs targeting glioblastoma were limited to cross the BBB/BTB; however, using LIFU+MBs, diverse therapeutics showed significantly higher uptake, improved tumor control, and overall survival among different species. Here, we highlight the therapeutic approach of LIFU+MBs mediated drug-delivery in the treatment of glioblastoma.

## Introduction

**Figure 1:**
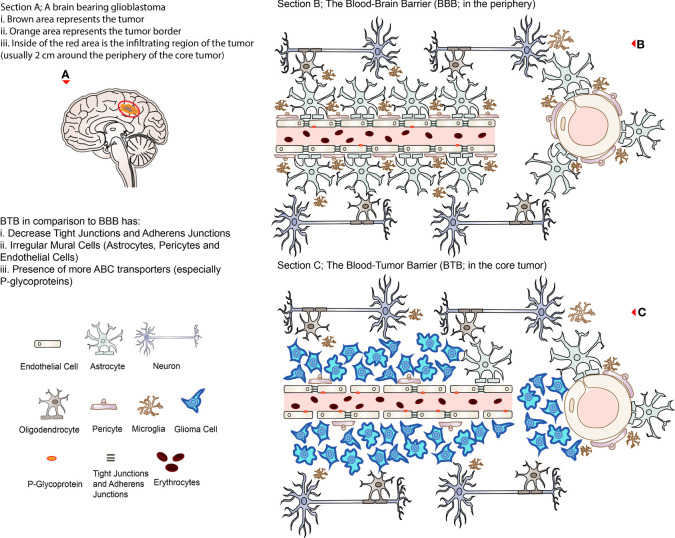
Representing illustrations of BBB and BTB. **(A)** A brain bearing glioblastoma (brown with orange border) with an invisible infiltrating region approximately 2 cm around the lesion (circled in red representing the region of recurrence after surgical excision). **(B)** The BBB in association with other cells present in the healthy brain microenvironment; the right side represents the cross-section of the cerebral vessels. **(C)** The BTB in the core of a glioblastoma; the right side represents the cross-section of the cerebral vessels. Note that TJs are disrupted, and ABC transporters (P-glycoprotein) are relatively increased in the BTB compared to the BBB.

Glioblastoma, the most common and primary brain tumor, accounts for more than half of total brain gliomas. It is a lethal and the most aggressive cancer of the central nervous system (CNS) with an overall poor prognosis (1, 2). According to the 2021 World Health Organization classification update, it is now termed as Astrocytoma, IDH-mutant (previously called Glioblastoma, IDH mutant) and Glioblastoma, IDH-wildtype; both of which are Grade IV high-grade malignant tumors (3, 4). For the sake of simplicity it will still be termed as “glioblastoma” in this review paper as most of the references are between the year 2017 to 2021 and precede the introduction of this new classification. These tumors are highly infiltrative and incurable (1, 5), affecting men more commonly than women, with very few risk factors identified so far (2). Since 2005, the treatment of glioblastoma has been limited primarily to surgical excision followed by radiotherapy and chemotherapy, which remains ineffective (2, 6), having a mortality rate of more than 90% in the first 5 years (2, 7). Despite the best multimodal treatment delivered, the risk of recurrence remains high due to infiltrating cells in the surrounding healthy brain parenchyma (8) (as shown in **Figure 1A**), which is commonly accompanied by severe neurocognitive sequelae and other neurological dysfunctions either due to the tumor itself or its related treatment (1, 9). In addition, total resection is unachievable due to glioblastoma’s invisible infiltrative and intractable nature (8, 9). In the recent past (October 2015), the Food and Drug Administration (FDA) approved the use of tumor treating fields, which is known to have antimitotic effects on rapidly dividing cancer cells, to be used as a fourth modality for the treatment of glioblastoma patients (10, 11), but as far as systemic treatment is concerned, there were no major improvements mainly due to the inability of therapeutics to cross the blood-brain barrier (BBB) (12).

The BBB, which was described over 100 years ago (in 1913 by Goldmann), is known to impede the delivery of macromolecular (>500 Da) up to 100% and micro-molecular drugs up to 98% from the peripheral circulation to the brain, including chemotherapeutics, thereby leaving the most novel and promising therapeutics unexploited (12–15). Despite the presence of the BBB, several techniques exist to deliver therapeutics in the CNS, either by bypassing or penetrating them (summarized in **Table 1**). These approaches can be categorized into: cellular, molecular, or physical/chemical (16). These techniques include convection enhanced delivery, trans-nasal delivery, direct intracranial and intra-arterial injections, osmotic and chemical disruption of the BBB, placement of Rickham/Ommaya reservoir, radiation therapy, direct delivery into glioblastoma sites during surgical excisions, and even re-engineering and chemical modifications of therapeutics (conjugating them to ligands which have a higher affinity for receptors/transporters expressed on the cerebral endothelial cells) to increase their uptake in the CNS (8, 12, 16–19). Despite being effective in various ways, these techniques are not routinely used in the clinic due to their drawbacks (12, 18, 19). These include high invasiveness, risk of hemorrhage/infections, heterogeneous distribution, low infusion rates and limited diffusion, non-local administration resulting in unwanted side effects to healthy brain tissues such as seizures, reflux of drugs from incision sites, permanent brain damage, and the difficulty of implementation if ever repeated administration is necessary. Therefore, glioblastoma treatment requires new strategies both in terms of systemic treatment and with better precision without affecting non-glioma tissues. Such an emerging technology that has facilitated therapeutics delivery both in the healthy brain and the unhealthy brain, including glioblastoma, is the usage of low-intensity focused ultrasound (LIFU) (19). This review paper addresses recent progress in the use of LIFU mediated drug-delivery for the treatment of glioblastoma mainly over the past 5 years.

**Table 1:** Current techniques to overcome BBB/BTB and their drawbacks over LIFU+MBs.

Techniques	Disadvantages over LIFU+MBs
Convection enhanced delivery (CED)	Invasiveness Risk of infection Risk of hemorrhage Low infusion rates and volumes Highly inconsistent distribution and tumor interstitial fluid pressure Rapid efflux of drugs from injection sites
Trans-nasal delivery	Limited capacity to selectively target brain regions Limited by the dosage volume that can be administered Difficulty obtaining proper alignment in the nasal cavity for effective delivery Risk of infection Risk of hemorrhage
Direct intracranial injections	Invasiveness Risk of infection Risk of hemorrhage
Osmotic and chemical disruption of the BBB	Globally transient disruption of the BBB Unwanted side-effects (such as seizures)
Intra-arterial injections	Systemic effect rather than localized BBB alterations Can induce complications such as neurologic deficits, seizures, and potential tumor migration
Radiation therapy	Exposure to ionizing radiation
Placement of Rickham/Ommaya reservoir	Invasiveness Risk of infection Risk of hemorrhage Possibility of tube blockage
Direct delivery into glioblastoma sites during surgical excisions	Invasiveness Not feasible to repeat
Re-engineering of therapeutics	Hurdles including first pass clearance Blood instability Immune response Off-target effects Lower level of drug extravasation

## Blood-Brain Barrier and Blood-Tumor Barrier and Its Heterogeneity

To maintain stable and ideal function of the healthy CNS, several demands, such as proper oxygen/nutrients supply and precise regulation of cerebral blood flow, are maintained by three known barriers (the blood-brain barrier, the blood-cerebrospinal fluid barrier, and the brain-cerebrospinal fluid barrier) (1, 20). The BBB is the most important barrier as it regulates the transport/exchange of materials between the blood and the brain while simultaneously restricting the passage of toxins, red blood cells, and pathogens acting as a defense mechanism (16, 20, 21). This selectively permeable brain interface anatomically comprises of cerebral endothelial cells (ECs) (which possess a series of physical, transport, and metabolic properties) connected by tight junctions (TJs), pericytes, astrocytic foot-processes, and others; all of which contributes to the barrier integrity (1, 16, 22). These three types of cells (ECs, astrocytes and pericytes), which are often connected to nerve-endings and microglias (the resident immune cells of the brain), are commonly also referred to as the neurovascular unit (NVU) (16, 21).

The NVU has a unique way of controlling the movement of hormones, ions, molecules, or cells either inside or out of the CNS which are very specifically regulated and referred to as the ‘gatekeeper’ for the CNS (16, 22). ECs, the very first obstacle in the BBB (the wall of the blood vessel, which accounts for the greatest surface area of the BBB), are held together by TJs that work symbiotically with adherens junctions (AJs) creating a strong bond to regulate paracellular, transcellular, and enzymatic crossing of substances (1, 16, 21). These TJs are made up of several proteins, including occludins, claudins (mainly claudin-5), junctional adhesion molecules (JAM), zonula occludens proteins (ZO-1, ZO-2), and associated molecules while cadherin-catenin complexes mainly form AJs (21). ECs are generally non-fenestrated, lacking the expression of E-selectin and intercellular cell adhesion molecule 1 (ICAM1) that prevents the leukocyte attachment and influx (1, 22). Moreover, there are the presence of receptors and channels such as ATP-binding cassette transporters (ABC efflux transporters) on both the luminal and the abluminal sides of the capillaries, which actively efflux substances out of the CNS (16, 23). These most commonly include ABCG2/BCRP, ABCB1/MDR1, ABCC1, ABCC4, ABCC5, ABCA2, and ABCA8 which can egress a large variety of lipid-soluble molecules (15, 21). This could explain why cerebral ECs contain significantly higher amounts of mitochondria compared to other tissues (22).

On the abluminal side, pericytes play a significant role in the regulation of vascular function (for instance, regulation of capillary diameter by their contractile proteins and angiogenesis) as well as expression of transporters, where its deficiency (in mice) displayed increased BBB permeability to both low and high molecular weight (MW) substances (16, 20, 22). They are usually wrapped around the ECs, where basal lamina is produced to attract end-feet processes of astrocytes (16). Astrocytes are the most common cell type found in the brain having their end-feet covering more than 90% of capillaries (on the abluminal surface), providing a link between neurons and blood vessels (16, 22). With the help of TJs and gap junctions’ connection, in association with basal membranes, these astrocytes can form glia limitans perivascularis which provides additional barriers (16, 21).

On the other hand, the barrier between the newly synthesized microvasculature in a tumor microenvironment and its adjacent expanding tumor, which is formed due to the increased nutritional/oxygen demands, is termed the blood-tumor barrier (BTB) (1). While the tumor is rapidly proliferating, it compresses the existing vasculature, stimulating the secretion of VEGF (in the hypoxic zone) and triggering angiogenesis-related genes such as hypoxia-inducible factor 1α (HIF1α) (15, 16). This results in angiogenesis (to ensure adequate nutritional/oxygen supply) where abnormal new vessels are synthesized, which are relatively more tortuous as well as heterogeneous and leaky (15, 16). These neo-barriers are modified versions of the original BBB, which have several altered properties compared to the latter (**Box 1**), such as a decrease or loss of TJs (including their respective proteins such as ZO-1, claudin-3, claudin-5, and occludins), irregular mural cells (pericyte, astrocytes, and microglia) distribution and disruption of astrocytic end-feet which further compromises the barrier integrity (1, 15, 16).

Box 1 **Properties of BTB limiting permeability.**

- Efflux transporters
- Heterogeneity of BTB
- Edema
- Increased interstitial pressure
- Dense extracellular matrix

Despite being characterized as leaky, the BTB has the equivalent capability of restricting drugs to variable extents, ranging from the core of the tumor to its periphery, where the BTB has the highest permeability compared to the latter (1, 15). This is mainly due to the heterogeneity of the BTB throughout the different pathological layers of the tumor, where both the integrity and function of the BBB are maintained in the periphery (and surrounding infiltrating cells) compared to the core tumor site (15, 16). Unfortunately, these differences in the permeability profiles result in an uneven distribution of therapeutic drugs in the tumor lesions such as glioblastomas, hence affecting the efficacy of the treatment (16, 23, 24). This is a consequence of the heterogeneous dysregulation of several transporters (especially ABC transporters; where P-glycoprotein is the most common), receptors as well as angiogenesis pathways and extracellular matrix (ECM) components in the NVU, where the expression of efflux transporters are more or less maintained or even upregulated in the BTB (15, 16, 25). In addition, there are dense ECM (26), edema, and increased interstitial pressures which are accumulated in the tumor site due to leaky and dysfunctional vessels that can further hinder therapeutic delivery (1, 16). This phenomenon has been observed in both adult and pediatric brain tumors (16). Altogether, it sums up to a BTB displaying properties such as higher efflux, modified transporter activities, and refined fluid dynamics (15). Overall, BBB and BTB create a boundary between the brain and the bloodstream protecting the brain microenvironment by restricting both endogenous and exogenous substances that can be potentially toxic, including immune cells and most of the systemically administered therapeutics making intracranial treatment difficult and challenging (12, 15). **Figure 1** illustrates the representation of the normal physiological BBB and BTB in the glioblastoma situation.

## Low-Intensity Focused Ultrasound

Ultrasound, which has a frequency higher than human hearing (>20 kHz), is one of the basic methods extensively utilized for diagnostic imaging and therapeutic purposes in our daily lives (27). Besides being non-invasive, it is readily available for real-time diagnosis and bears a low cost (28). Since its discovery, it has evolved from being one of the safest diagnostic tools, to a cost-effective therapeutic tool; initially with thermo-ablation and now with its ability to open temporarily the BBB/BTB (19, 29). Focused ultrasound (FUS) can be classified as high-intensity focused ultrasound (HIFU) and low-intensity focused ultrasound (LIFU). Both make use of a special concaved transducer, lens, or phased array to converge the ultrasonic waves (which have been converted from electric signals) into a small and precise volume of tissue at the center (focus) (19, 27). **Table 2** outlines some differences between LIFU and HIFU. This energy is usually delivered in a 3-dimensional space within the brain/tumor tissue; a targeted treatment volume, where the power is highest at the center compared to its non-targeted tissue (outside the focus) (18). Thermo-ablation (by HIFU), which is used for tissue destruction and necrosis, is usually an application of continuous waves of ultrasound (producing thermal effects). In contrast, LIFU uses relatively lower energy pulsed waves (i.e., non-continuous), which is focused on exogenously administered microbubbles (MBs) to temporarily disrupt BBB/BTB (*via* mechanical effects) by their additive power at the focus point (15, 18). In this way, intraveneously administered onco-therapeutics for the treatment of glioblastoma are allowed to cross the disrupted BBB/BTB in a given time frame with relatively less effort until its restoration (closure). Using LIFU, various onco-therapeutics can be used for exploitation in glioblastoma treatment in contrast to HIFU which is mainly used to destroy the glioblastoma tissue and its surroundings. Intermittent FUS has proven to have greater BBB/BTB disruption in tumor tissues compared to continuous FUS (30). During the intervals in an intermittent application (determined by duty cycle) of ultrasonic energy, the MBs have the time to circulate from bigger vessels to the small capillaries where the transducer is being targeted (30). Unlike HIFU, LIFU results in a minimal increase in temperature causing almost no harm to adjacent normal brain parenchyma when the correct set of parameters is applied (15, 18). Application of LIFU+MBs can result in a variety of biological and chemical effects that have been reported to be non-invasive, precise, reversible, repeatable, and controllable both in deep and superficial lesions (15, 19). These are major advantages of LIFU+MBs (**Box 2**) over the current available techniques that are used to overcome the BBB; it has been discussed earlier in the Introduction and summarized in **Table 1**. Currently, one of the most extensively explored applications of LIFU+MBs is the temporary BBB/BTB disruption followed by the delivery of therapeutics in the LIFU targeted zone (discussed in later section). This incisionless technology is a promising tool that can substitute several existing procedures in several CNS disorders, including glioblastoma, while allowing a more uniform distribution of therapeutics throughout the heterogeneous tumor microenvironment especially in critical regions for surgical interventions, such as the brainstem (15).

**Table 2:** Comparison of LIFU and HIFU.

LIFU	HIFU
Lower energy needed	Higher energy required
No harm to tissue; facilitates drug delivery	Destructive effect; thermo-ablation (no drug-delivery)
Minimal increase in temperature	Thermal effects
Intermittent wave (non-continuous)	Continuous wave application
Requires MBs	MBs not required
Higher penetrance of ultrasonic waves (due to the lower frequency)	Lower penetrance of ultrasonic waves (higher attenuation due to longer wavelength)

Box 2 **Characteristics of LIFU mediated BBB/BTB disruption.**

- Immediate
- Non-invasive (incisionless)
- Precise
- Repeatable
- Controllable
- Reversible
- Safe
- Minimal temperature increase

### Mechanism of LIFU Mediated BBB/BTB Disruption

BBB/BTB disruption mechanism after applying LIFU+MBs is still debatable, where a combination of mechanical and functional changes (**Box 3**) appears progressively with time and disappears in the following hours/days (8). After intravenous administration, when these tiny MBs circulate in the blood vessels and eventually enter the specific zone targeted by the LIFU transducer, they undergo several kinds of behaviors, mainly due to the high compressibility of the entrapped gas compared to the surrounding fluid present in that particular area (12). These include expansions and contractions during the compression and rarefaction phases of the ultrasound pressure wave, respectively, as well as oscillations that require energy of lower magnitude compared to HIFU (12, 31). These behaviors, such as oscillations, are often described as acoustic cavitation or simply cavitation (31–33). Cavitation can be classified as either stable or inertial, dependent on the acoustic pressure applied (12, 19, 33). MBs oscillations are rather stable, linear, and symmetric at low ultrasonic pressures generating acoustic emissions at harmonics of the driving frequency while an increase in pressure leads to non-linear oscillations generating subharmonic and ultra-harmonic emissions (12, 32). Increasing this pressure further leads to unstable oscillations of the MBs (inertial cavitation) where overexpansion and violent collapse/disruption of MBs can produce undesired shockwaves, micro-jets, and mechanical and thermal stresses that can be distinguished by wideband frequency emissions (12, 19, 34). The effect of cavitations on the BBB/BTB can be defined either by a mechanical index; MI (which is the negative acoustic pressure divided by the square root of the frequency) or cavitation index (CI, which is the negative acoustic pressure divided by the frequency) (15, 27). These indices are used to assess the LIFU+MBs mediated BBB/BTB opening where both correlate to the degree of disruption, assessing either their mechanical bio-effects or the MBs cavitation activity, respectively (31).

Box 3 **Mechanisms involved in LIFU mediated BBB/BTB disruption.**

- Stress on ECs
- Stretching of cerebral blood vessels
- Elevation of EC temperature
- Opening of TJs
- Altered protein expressions in ECs
- Increase in trans-endothelial fenestrations
- Increased formation of caveolae
- Regulation of TJ integrated adhesion molecules (claudin-1, claudin-5, occludin, and ZO-1)
- Release of α2-macroglobulin by NVU
- Decrease of P-glycoprotein (drug efflux transporter)

**Figure 2:**
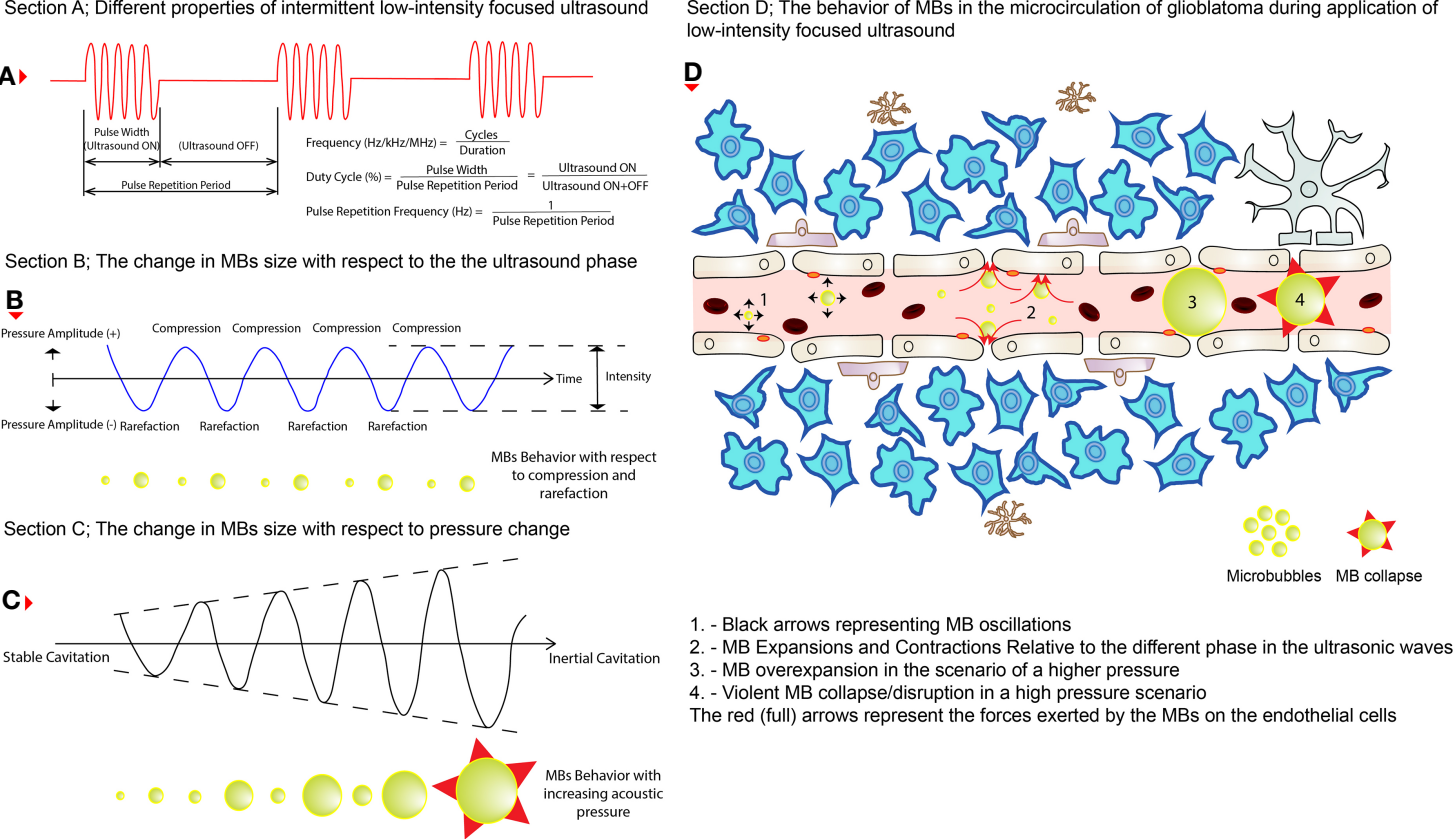
The behaviors of MBs during LIFU application. **(A)** The different properties of an intermittent pulsed LIFU. **(B)** How MBs change size (shown below the wave) during the two phases of the ultrasound (i.e., compression and rarefaction). **(C)** How do MBs increase in size (shown below the wave) while pressure is increased eventually resulting in violent collapse? **(D)** How do these changes affect the permeability of the cerebral vasculature in glioblastoma tissues? The black (short arrows) arrows represent oscillations on the left, expansions and contractions in the middle, and eventually collapse on the right of the illustration. The red (full) arrows represent the pressure exerted by the MBs on the ECs of the cerebral blood vessels.

The constantly changing morphology (shape and size) and the oscillation of the MBs in the cerebral microvasculature during LIFU application causes fluid streaming around them, which in turn results in shear and circumferential stresses being exerted on blood vessel walls (i.e., ECs) (12, 34, 35). Furthermore, the MBs expansion may cause stretching of the blood vessels which may transiently open tightly sealed junctions in the BBB/BTB (28). This opening can also be due to the oscillatory push-pull action of the MBs on the ECs (31). One more hypothesis suggests that elevation in EC temperature can alter protein expressions in ECs thereby increasing its permeability (36). Collectively, all these actions result in a temporary BBB/BTB disruption, leading to an upregulation in trans-cellular and para-cellular transport of molecules across the BBB/BTB (15, 19), which can also be addressed as sonopermeation (permeability due to ultrasound) (32). This can happen due to various mechanisms on the molecular level proposed by diverse preclinical studies such as disruption of TJs, increase in trans-endothelial fenestrations, and increased formation of caveolae (8, 19, 37). Application of LIFU+MBs has been shown to regulate several TJ integrated adhesion molecules such as claudin-1, claudin-5, occludin, and ZO-1 (31, 38). Another possible explanation is that substances, such as α2-macroglobulin, are released by the NVU after LIFU+MBs to protect its integrity whereby BBB/BTB is further disrupted (39). Additionally, the most dominant protein responsible for drug efflux, P-glycoprotein expression, is decreased for up to 48 h post sonication (8, 19, 40, 41). Besides, there is also an increase in K_Ca_ (calcium activated K^+^ channels) after LIFU+MBs application in gliomas, which plays an essential role in transcellular permeability in BBB/BTB (30). The advantage of using this technique is that the integrity of the BBB/BTB begins to restore almost immediately after the disruption itself and can be completed within the upcoming 6-24 h (8, 42). Perhaps, this can be classified as a drawback of using this technique as the procedure needs to be repeated for the next treatment session. But on the other hand, it also holds the advantage of being repeatable. It makes it easier to counter this disadvantage. In short, LIFU+MBs leads to mechanical effects, which can increase the permeability across the BBB/BTB. This is a beneficial approach that is being intensely investigated nowadays in therapeutics delivery (32). **Figure 2** illustrates the mechanism of action of MBs upon the application of LIFU.

### Microbubble

Microbubbles, which are commonly encapsulated with gas, have been in diagnostic use and treatment for many years (29). They are used as contrast agents to assess blood flow *via* ultrasound (12). These gases are usually perfluorocarbon with an outer lipid shell (34). In some cases, the components can be proteins, liposomes, inorganic, or polymers resulting in different physiochemical properties (29, 34). The three main MBs that have been utilized in preclinical studies are both FDA and EMEA approved for diagnostic usage (8, 43). These include Luminity/Definity (by Lanthus Medical Imaging), Optison (by GE Healthcare), and SonoVue/Lumison (by Bracco Diagnostics) (8, 31). Each has different concentrations, half-lives, and hydrodynamic sizes (8, 31). While all of them produce the same effect on BBB/BTB disruption, smaller MBs have been associated with less permeability and shorter recovery period of the BBB/BTB disruption (31). In the same way, the concentration of MB used is directly proportional to the magnitude of the BBB/BTB disruption produced, where a higher concentration will result in significantly larger BBB/BTB disruption (31). Novel research has led to the engineering of MBs in innovative ways, where they can be encapsulated or even conjugated with therapeutic drugs and targeting ligands that can further improve their circulation time while having better/specific targeting (29, 31, 44). In addition to these traditional tiny MBs, there is now research using tinier bubbles on the nanoscale level (nanobubbles, nanodroplets, and nanocomposites) (32, 45). Nanodroplets have a liquid perfluorocarbon core which increased the circulation time to over half an hour compared to the former MBs used (44). Furthermore, these nanodroplets can be engineered so that the encapsulated substance can be converted from liquid to gaseous phase *via* laser activation (46). On the other hand, nanobubbles have been designed in a specific way where active image tracking is possible into the deep glioblastoma tissues (45). These nano-scale bubbles have stronger penetration power and enhanced permeability and retention effect (EPR) (29). The use of MBs and their cavitation effect during LIFU application allows ultrasound of lower energy to be used to achieve BBB/BTB disruption, which can be at least three orders of magnitude lower than that used in thermo-ablation (19, 34). Without the use of MBs, there is usually no disruption of the BBB/BTB (47).

### Parameters (Factors Influencing LIFU Mediated BBB/BTB Disruption)

The magnitude of BBB/BTB disruption for therapeutic delivery in a safe and optimum manner depends on several parameters (**Box 4**). Different literature has different sets of parameters to induce BBB/BTB disruption. These discrepancies hamper the consensus on the optimal parameters needed while making a comparison of the results obtained more difficult throughout different studies (15, 32). Perhaps the first factor responsible for this variability is the type of transducer utilized to generate the ultrasonic waves (28). The second set of parameters is the skull’s impedance to LIFU penetration (28, 48, 49). As the thickness of animal models is not similar to human beings, it is difficult to reproduce the same preclinical effects in the clinic (50). Unlike electromagnetic waves, ultrasound requires a medium for its proper propagation (27). Moreover, these waves are subjected to attenuation, reflection, amplification, absorption, and scattering (51, 52). As the bone is irregular and varies in thickness and density, it strongly attenuates, reflects, and distorts ultrasound to variable extent (15, 19). In the past, the utility of HIFU for thermal ablation was greatly attenuated due to its longer wavelength (12). But as the frequency is lowered (such as in LIFU), the penetrance increases (27). Third, the presence of hair can distort the delivery of ultrasound by up to 80%. This is because hair introduces typically air in the path that ultrasound needs to propagate (19).

Box 4 **Factors influencing BBB/BTB disruption.**

- Frequency
- Pressure amplitude
- Power
- Duration
- Burst parameters (burst repetition frequency)
- Duty cycle
- Microbubble type, size, dosage and content
- Cranium impedance
- Type of transducer
- Mechanical index
- Cavitation index
- Presence of hair
- Mechanical Index
- Cavitation Index
- Presence of hair

In addition, acoustic pressure (measured in MPa), the ultrasound frequency used (measured in Hz), pulse duration (measured in ms), burst pulse repetition frequency (measured in Hz), duty cycle (percentage time the ultrasound is on), exposure duration (measured in minutes), MB type, size, dosage, and content are all parameters that play equivalent roles that can influence the permeability of BBB/BTB (12, 15, 19, 27, 34). Most of the LIFU mediated BBB/BTB disruption in glioblastoma has been achieved with a range of frequencies around 1 MHz (refer to **Table 3**). Talking about pressure, in the range of 0.31 to 0.84 MPa, the application of different acoustic pressure results in different permeability of molecules, which increases in order of their respective molecular size and weight with increasing acoustic pressure (34, 57), where molecules of up to 2000 kDa/54.4 nm (Dextran) have safely been delivered (19). Below this range (for instance, at a pressure of 0.28 MPa), the uptake of nanoclusters was negligible in glioblastoma, while having no significant difference at 0.61, 0.72, and 0.85 MPa, respectively (60). Recently, it has also been shown that with increasing pressure, the diffusion of water molecules increases due to the upregulation of aquaporin-4 (75). Moreover, these parameters also determine whether the therapeutics uptake will be early/fast or slow/late (8). Other factors such as the introduction of TJ protein binders (claudin-5 binder) can greatly facilitate the BBB/BTB disruption which can be more effective and safer (76). It is important to carefully investigate and pick the appropriate set of parameters as the correct parameters can yield in BBB/BTB disruption that can be reversible. In contrast, incorrect selection of parameters can produce irreversible injury to the brain tissue (discussed in a later section) (12).

**Table 3:** Parameters and MBs used in different preclinical studies.

MB	Frequency	Pressure	Duration	Duty cycle	Pulse repetition frequency	Ref
SonoVue/definity	1.5 MHz	0.7 MPa –acoustic pressure	2 min		5 Hz	(53)
Polydisperse in-house manufactured	1.5 MHz	0.7 MPa – peak negative pressure	30 sec		5 Hz	(54)
Definity	1.78 MHz		3 min		0.67 Hz	(55)
SonoVue	1 MHz	1 W – acoustic power	1 min		1 Hz	(38)
SonoVue	500 KHz	0.63 and 0.81MI	2 min		1 Hz	(56)
Albumin-shelled MBs	1 MHz	0.45, 0.55 MPa peak negative pressure	2 min	0.50%		(57)
Lumason	1 MHz	0.3 MPa acoustic pressure measured in water	2 min		1 Hz	(58)
Definity		0.68-165 KPa peak negative pressure	55 sec		1.1 Hz	(59)
In-house prepared	1.5 MHz	0.61, 0.72, 0.85 MPa	1 min	3.33%	5 Hz	(60)
Definity	1.1 MHz	0.85 MPa peak refractional pressure in water	2 min			(61)
Softshell BG8235 (Bracco)		0.28-0.55 MPa	3 min		1 Hz	(62)
SonoVue	1.05 MHz	0.3 MPa acoustic peak pressure	2 min		1 Hz	(63)
In-house prepared	0.996 MHz	0.64 MPa peak rarefactional pressure	1 min	30%	1 Hz	(64)
In-house prepared	1.1 MHz	0.32 MPa *in situ* pressure	6 min		1 Hz	(65)
Definity			75 sec		1.1 Hz	(47)
Definity	1.68 MHz	0.25 MPa starting pressure with increments of 0.025	2 min		1 Hz	(66)
In-house prepared		1.84 W power				(67)
Definity	1.68 MHz		2 min		1 Hz	(68)
	1 MHz	0.3 MPa acoustic pressure	1 min	1%	1 Hz	(69)
	1 MHz	0.30 MPa peak negative pressure	1 min			(70)
SonoVue	1.0 MHz	2.86 W power	1 min	5%	1 Hz	(71)
In-house prepared	1.7 MHz	1.3 mechanical index	10 min			(30)
In-house prepared	1 MHz	0.5-0.9 MPa acoustic pressure	2 min		5 Hz	(72)
Self-prepared albumin shelled MB	1.14 MHz	0.60, 0.80 MPa peak negative pressure	2 min	0.5%		(26)
In-house prepared	1.1 MHz	0.64MPa peak refractional pressure	1 min		1Hz	(73)
Definity		0.68-0.72 MPa	1 min		1Hz	(74)

## BBB/BTB Disruption and Evaluation

**Figure 3:**
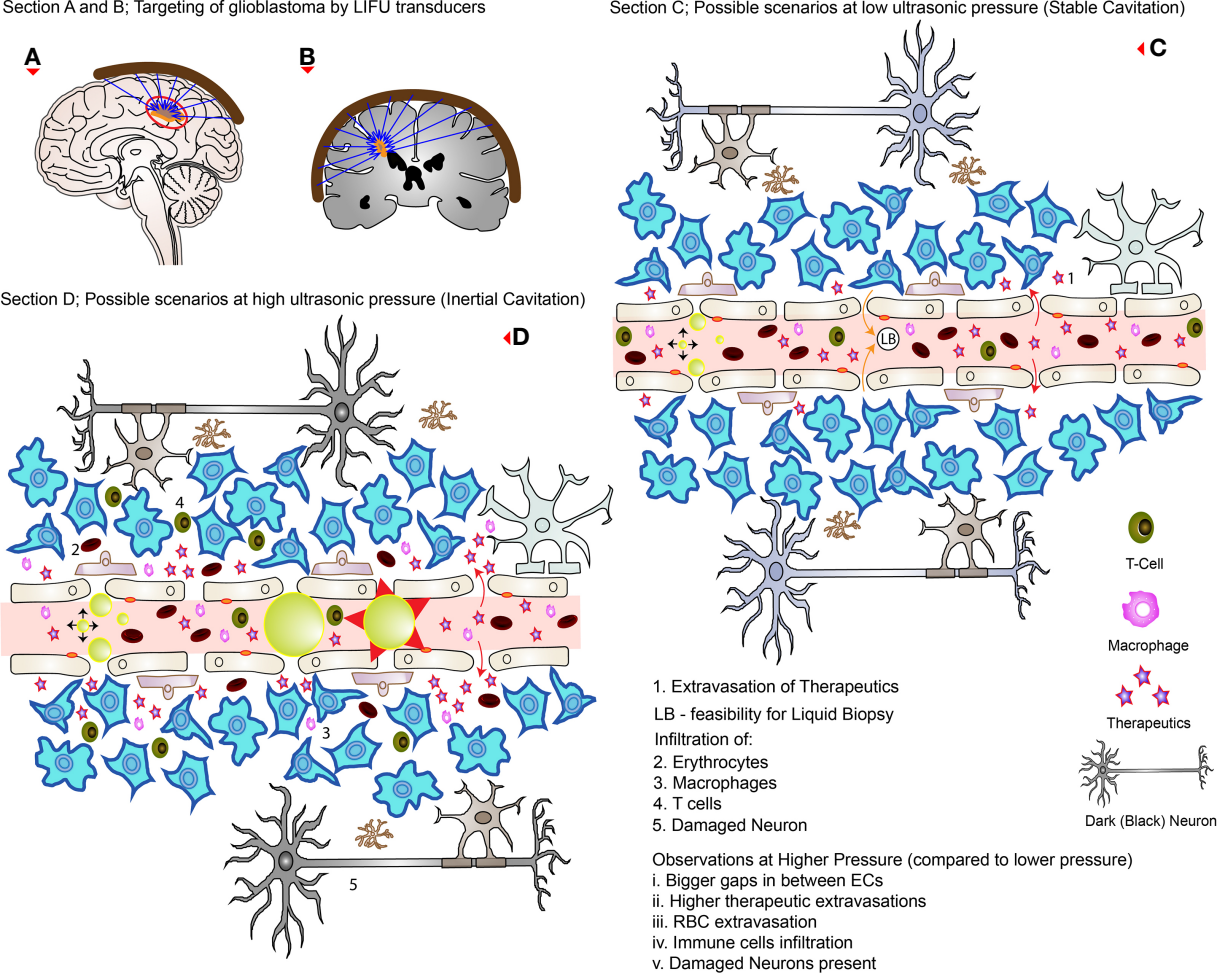
LIFU targeting and its effects during high and low pressures. **(A, B)** How external transducers (around the brain) target glioblastomas in practice by the converging ultrasound waves (in the direction of the blue arrows). **(C)** The possible scenarios at low pressure (stable cavitation); the RBC, and immune cells are confined to the blood vessels while disrupting TJs and creating openings with low levels of therapeutic extravasation. **(D)** The possible scenarios at higher pressure (inertial cavitation) where there is greater concentration of drugs delivered with wider openings between the ECs. Note that there is MB collapse, immune cells, and RBC extravasations as well as affected neurons (which appears dark on analysis). The red arrows (curved full arrows) represent drug extravasation while the orange arrows (curved full arrows) represent the feasibility of liquid biopsies.

In practice, the aperture of the converging LIFU by the transducer is usually very small and precise. This can be inefficient to target a large volume of tissue, leaving behind the surrounding parts unsonicated. Therefore, many studies have developed their own targeting method to cover the whole glioblastoma tissue volume and sometimes the infiltrating regions or even a whole hemisphere in some cases. These are summarized in **Table 4**, where many studies used a grid system to target four specific points. **Figures 3A, B** show how LIFU can be applied to a certain location in the brain where all the waves merge to produce the additive effect. Alternative to multiple targeting points, neuro-navigation has been attempted in several studies to precisely target these lesions without the need for pre-treatment MRI scanning (78–80). Following this procedure, either with a single sonication point or with several sub-spots targeting, confirming the success of BBB/BTB disruption is an essential step in glioblastoma treatment studies. There are several ways to investigate it. The most common ways are the use of contrast-enhanced magnetic resonance imaging (CE-MRI) (81) and dye leakage, in particular Evans Blue (EB) (82–84), which has been utilized in several preclinical studies. The former is a less invasive technique that requires the administration of an image tracer (which is usually impermeable to BBB/BTB) while the latter usually requires the termination of the animal used and macroscopic visualization with the naked eye. EB can also be visualized under the microscope with the correct excitation and emission wavelength (30, 63, 70). In addition to EB (which has a longer circulation time), FITC-Dextran has also been proposed, which has a higher MW and shorter circulation time where their combined usage can be even more beneficial in quantifying the amount of leakage in the brain parenchyma (82). Trypan Blue (74), Sodium Fluorescein (58, 73), and Nile Blue (44) are other alternatives that can be used instead of EB. As far as image tracers are concerned there is a variety of them, namely Gd-DTPA (diethylenetriamine penta-acetic acid), superparamagnetic iron-oxide (SPIO) NPs, horseradish peroxidase, lanthanum chloride, ionic manganese, Alexa Fluor 488, Texas-Red-tagged dextran, GFP-tagged dextran, gold nanorods, and ^99m^Tc-DTPA (31, 83, 85). These tracers can be visualized either by *in vivo* imaging or other different ways of imaging techniques under the microscope (31). Other emerging techniques include diffusion tensor imaging (86), bioluminescent imaging for drug uptake (refer to **Table 5**), and PET imaging (39, 60, 61, 77, 83). Recently, apart from the existing glucose, mannitol, and inulin derivatives, [^18^F]2-fluoro-2-deoxy-sorbitol (18FDS) has also been proposed for PET imaging (83). Another newly used technique in preclinical studies is the change in K_trans_ values, which is short for transfer coefficient from blood to brain extravascular space (87). This is also a non-invasive technique to determine the permeability, which correlates strongly to EB and Gd-DTPA extravasation in glioblastomas (30, 56, 74). Its value has been demonstrated to have a linear relationship with drug extravasation and concentration in glioblastoma tissues (74). Another recent study showed the V_e_ map’s feasibility to assess BBB/BTB permeability (56). Additionally, the counterpermeability (K_ep_), the permeability from the extravascular-extracellular space (EES) toward capillaries can also be monitored, where the imbalance in the K_trans_/K_ep_ ratio reveals the possibility of enhanced drug retention in the EES for drug delivery (88). Despite being non-invasive, MRI does not allow real time tissue sampling (58). Therefore, many studies implemented the use of passive cavitation detection (PCD) which can be used in real time to monitor the acoustic emissions of MBs (54, 80, 89). It can help to show if the cavitation produced is either stable or inertial. **Table 5** summarizes the different preclinical studies using MRI, contrast agents, tracer dyes, PCD, quantification techniques, *in vivo* imaging, and some of their purposes in the particular study.

**Table 4:** Targeting strategies utilized by different preclinical studies to cover the whole tumor volume or its infiltrating volume or sometimes a whole hemisphere.

Application of LIFU	Ref
Applied once at 4 points on a 2 mm-by-2 mm grid	(53)
Applied once at 4 points on a 1.5 mm-by-1.5 mm grid	(54)
9 points targeting grid spaced 1 mm apart	(55)
8 target spots	(57)
Mechanical zig-zag shaped scan (XY-axis) to cover a square of 6 mm-by-6 mm	(77)
36 overlapping targets to cover most of the cerebrum	(59)
Transducer focused *via* 3-point triangulation	(60)
Sonication volume consisting of 10-20 target points	(61)
4 sonication targets in a 2x2 matrix distanced 1.5 mm apart	(65)
27 locations for LIFU application	(47)
4 points overlapping grid	(66)
2 sites of sonication with 2-mm gaps in between	(72)
9 spots on a 3 mm-by-3 mm square grid	(26)
5 targets in and around the tumor	(74)

**Table 5:** The purpose of using imaging, quantification of therapeutics, and monitoring of MB activity in preclinical studies.

MRI	Contrast agent	Dye	LC-MS/MS	IVIS	PCD	Ref
To check tumor location/size To assess BBB permeability	Gd-DTPA	EB	To measure etoposide concentration in the intracranial tumors		Yes	(53)
Investigate tumor progression Evaluate BBB opening	Gadodiamide		To measure etoposide concentration in the intracranial tumors		Yes	(54)
Tumor localization pretreatment Assess BBB permeability	Gadovist	EB	Determination of Dox levels in tissue after sonication			(55)
		EB		Yes		(38)
To assess kinetic change in BBB permeability by DCE-MRI	Gd-DTPA					(56)
In combination with LIFU To assess BBB opening For transport analysis	Gadolinium contrast agent			Yes	Yes	(57)
		NaFl	Measure PTX in plasma and brain			(58)
In combination with LIFU Check tumor location Evaluate BBB permeability	Gadavist		HPLC to measure irinotecan plasma and tissue concentrations		Yes	(59)
In combination with LIFU	^68^Ga-DOTA-ECL1i radiotracer					(60)
In combination with LIFU Assess tumor size Assess BBBO	Gadobutrol, Gadovist					(61)
Coupled to LIFU Measurement of tumor size Confirmation of BBB Opening	Gd-DOTA		Drug quantification in serum		Yes	(62)
		EB	UPLC coupled with MS/MS to quantify drug in plasma and brain	Yes		(63)
				Yes		(64)
In combination with LIFU To evaluate tumor development To evaluate BBB Opening	Omniscan (Gd-contrast agent)		To quantify delivery of Cabazitaxel			(65)
Evaluate BBBD	Gadavist		Measure concentration of drug in tissue and plasma samples			(47)
In combination with LIFU Confirm BBB Opening	Gadovist	EB	Dox quantification			(66)
Monitor therapeutic effect		EB		Yes		(67)
In combination with LIFU To confirm BBB Opening	Gadovist		ICP-MS for platinum (for cisplatin) and gold content quantification	Yes		(68)
Evaluate tumor location		EB		Yes		(69)
		EB		Yes		(70)
Verify tumor progression			HPLC to quantify Dox concentrations in tumor ECF and plasma			(71)
Measure BBBO	Omniscan	EB				(30)
		EB				(72)
In combination with LIFU Assess tumor size and invasiveness						(26)
Check tumor progression		EB, NaFl	HPLC to quantify drug concentrations in organs and plasma	Yes		(73)
For brain target selection, Characterize BBBD/tumor Evaluate brain tissue damage	Gd-DTPA	TB				(74)

LC-MS/MS, liquid chromatography with tandem mass spectrometry; IVIS, spectrum in vivo imaging system; PCD, passive cavitation detection; Gd-DTPA, gadolinium-diethylenetriamine pentaacetic acid; Gadovist, gadolinium contrast agent; DCE-MRI, dynamic contrast enhanced MRI; NaFl, sodium fluorescein; Gadavist, gadolinium contrast agent; ^68^Ga-DOTA-ECL1i radiotracer, low molecular-weight, short lived radiotracer; Gadobutrol, gadolinium contrast agent; ICP-MS, inductively coupled mass spectroscopy; TB, Trypan blue; HPLC, high performance liquid chromatography; UPLC, ultra performance liquid chromatography.

## Therapeutic Strategies Exploiting LIFU+MBs Based Targeting

### Drug Delivery for Glioblastoma

Delivering drugs to glioblastoma has been a nightmare mainly due to the presence of the BBB/BTB as well as the characteristics of the therapeutics such as their size and MW (34). Even the bioavailability of small MW drugs such as temozolomide (TMZ) is typically low in the target glioblastoma tissues compared to the plasma concentration levels (34). Due to this reason, there is a need for its frequent and continuous administration, which is associated with the risk of systemic side-effects, without forgetting the higher cost involved (19, 34). On the other hand, even after continuous administration, there is still no guarantee of better treatment efficacy. The heterogeneity of the BBB/BTB can make the distribution and concentration of these therapeutics uneven, thus leading to incomplete treatment of glioblastomas (34). Following LIFU+MBs mediated BBB/BTB disruption, these problems can be tackled by delivering a higher concentration of therapeutics in targeted areas, which can be controlled by the magnitude of the BBB/BTB disruption (8, 34). Using this approach, delivery of several anti-cancer molecules has been investigated in various brain disorders including different models of glioblastoma. These include antibodies, enzymes, neurotropic factors, genes, DNA, viruses, cells, immune therapeutics, nanoparticles, and mainly chemotherapeutic drugs which have different MWs, in particular, trastuzumab, doxorubicin (Dox), TMZ, methotrexate, carboplatin (Car), carmustine, irinotecan, paclitaxel (PTX), bevacizumab, and IL-12 (34, 39, 85, 90, 91). **Table 6** summarizes the different therapeutics that have been delivered to mainly glioblastoma models in the recent 5 years.

**Table 6:** List of drugs delivered to experimental animals bearing different cell lines of glioblastomas in preclinical studies.

Organism	Cell line	Drug delivered	Fold increase	Platform	Tumor control	Increased survival	Ref
Mice	PDGF driven HGG	Etoposide	8x			Similar	(53)
Mice	MGPP3	Etoposide	8x		Yes	Yes	(54)
Mice	DIPG Cell Line	Dox	4x		Yes		(55)
Mice	C6-Luc	AMPTL		NP	Yes	Yes	(38)
Mice	U87/B16F1ova	Gene delivery	4x	NP			(57)
Mice	PDX (MES83/GBM12)	Paclitaxel	3x to 5x				(58)
Rat	F98	Irinotecan	1.8x to 4.6x		No difference	No difference	(59)
Mice	DF1 cells	^64^Cu-CuNC	2x	Nanocluster			(60)
Mice	PDX HGG	Antibody		^89^Zr-radiolabeled			(61)
Mice	SMA-497 Cell line	TMZ			Yes	Yes	(62)
Mice	U87/PDCL	Carboplatin	4.2x		Yes	Yes	(63)
Mice	U87	DVDMS	3.43x		Yes	Yes	(64)
Mice	PDX (P3)	Cabazitaxel			Yes		(65)
Rat	F98	Carboplatin	2.9x		Yes	Yes	(47)
Mice	Patient-derived DIPG cell lines	Dox	>50x				(66)
Rat		Cisplatin					(66)
Rat	C6	shRNA		Liposome	Yes	Yes	(67)
Mice	U251	Cisplatin	2-3.5x	Gold NP	Yes		(68)
Mice	U87	Dox	4x	HMONs	Yes	Yes	(69)
Mice	U87	PTX		PPNP	Yes	Yes	(70)
Mice	GBM8401	Dox	2.35x				(71)
Rat	C6	HSV-TK/GCV	3.8x (over CMB gp) & 1.9x (over direct injection gp)	VCMBs	Yes	Yes	(72)
Rat	9L gliosarcoma	Cisplatin	6x	BPN	Yes	Yes	(26)
Rat	F98	Cisplatin	28x	BPN	Yes	Yes	(26)
Mice	U87	PTX	2x	Liposome	Yes	Yes	(73)
Rat	9L gliosarcoma	Dox					(74)

PDGF, platelet derived growth factor; HGG, high grade glioma; MGPP3, murine glioma cell harboring Pdgf^+^, Pten^-/-^, and P53^-/-;^ DIPG cell lines, SU-DIPG-17; NP, nanoparticle; AMPTL, NP consisting of an endogenous reactive oxygen species-cleavable thioketal linkers conjugated to paclitaxel (PTX) and autophagy inhibitor 3-methyladenine, and angiopep-2 peptide modified DSPE-PEG_2K;_ PDX, patient derived xenograft; DF1 cells, virus producing cells expressing PDGF-B, H3.3K27M, and Cre (to delete p53 specifically in the tumor cells); ^64^Cu-CuNC, ultrasmall and biodegradable copper nanocluster intrinsically labeled with ^64^Cu; SMA-497 cell line, TMZ-resistant glioma; PDCL, patient derived cell line; DVDMS, sinoporphyrin sodium; PDX, patient derived xenograft; HMONs, hollow mesoporous organosilica NPs integrated ultrasmall Cu_2-x_Se particles; PPNP, polysorbate 90-modified paclitaxel-loaded PLGA NPs; GBM8401, human brain malignant glioma cells; HSK-TK/GCV, Herpes Simplex Virus type 1 thymidine kinase/ganciclovir; VCMBs, VEGFR2-targeted cationic MBs; CMBs, cationic MBs.

There are several observations that have been noted following LIFU+MBs mediated drug-delivery. By far, the most obvious one is a significant increase in the amount of therapeutics being delivered to the targeted brain/tumor parenchyma as compared to its non-sonicated controls [measured commonly by *in vivo* bioluminescent imaging and liquid chromatography-tandem mass spectrometry (LC-MS/MS)]. These observations were similar for glioblastoma in the cerebrum as well as in brainstem gliomas (DIPG; diffuse intrinsic pontine glioma) as shown in **Table 6**. Usually, less drug is needed to be administered using LIFU+MBs compared to non-sonicated situations (68). This increase of therapeutics in these sonicated tissues is attributed mainly to increased permeability (77, 92) in those particular areas and the down regulation of P-glycoprotein expression in the vasculature of glioblastomas which is known to be responsible for drug efflux mechanisms (14, 65, 70). Besides, there is also a decrease in JAM-A, which is noted after LIFU+MBs mediated BBB/BTB disruption (30). These changes can be observed as early as 10-15 min following the procedure which increases as time passes by (47, 59, 77, 93) showing a prolonged exposure (77), as well as retention and diffusion in targeted areas (38, 54, 71, 74), which is independent of the drug concentration (77). This elevated level of drug in sonicated tumors could still be seen later, even when the drug concentration in plasma has decreased (47). Additionally, there is also an increase in the brain tumor-to-serum ratio relative to controls (53–55, 59). In the case of gene delivery, the transgene expression was significantly increased in glioblastomas following LIFU+MBs procedure (57, 67, 72). Moreover, interstitial fluid transport was seen to be augmented with an average of two-fold increase in flow-velocity magnitude (57).

### Safety and Toxicity

Many authors have investigated the safety of LIFU+MBs mediated BBB/BTB disruption protocol in preclinical studies with no long-term complication reported to date (19, 85). With the use of optimal parameters, these procedures have been shown to be well tolerated in various experiments using both mice and rat models bearing glioblastoma, with no toxicity (53, 59, 63, 66, 70). There was no evidence of hematological toxicity, spleen damage, liver, renal, and myocardial dysfunction where major blood components (RBC, WBC, and platelets) stayed within normal ranges (38, 62, 69). Results were similar both in the short term (4 h) and long term (14 days) (66). These investigations revealed no change in vital signs such as heart rate and respiratory rate (53, 66) as well as in animal weight (53, 59, 66, 69, 94). Motor coordination and cerebellar functions were unaffected as well (53, 66) with normal behavior post-treatment (68, 94). On histologic analysis, if the acoustic pressure was not too high (i.e., optimum), sonicated brain and non-sonicated brain, and glioblastoma tissue appears to be similar (47, 59, 74) showing no signs of pathological changes (30, 63, 69, 70, 92, 94), including parenchymal injury, necrosis, or micro hemorrhage (38, 53). There was no damage in brain tissue regardless of high or low doses of therapeutics delivered compared to controls (38). Even the use of MRI as a source of investigation revealed no tissue damage (92). There was no change in the neuronal number and no significant changes in apoptosis (66). Multiple sessions of LIFU+MBs, either as a sole treatment or in combination with different therapeutic drugs, were all well tolerated without evidence of any sort of brain tissue damage (19, 47, 58). These changes are normally reversible without harming the BBB/BTB if the ultrasound pressure is within the optimum range (62). When the acoustic pressure exceeds the optimum range (0.31-0.84 MPa as discussed earlier), for instance, 0.85 or 0.90 MPa, erythrocytes extravasation (petechiae) can be detected (60) which increases with increasing pressure, respectively (72). This is an indication of vascular damage (59, 74). If taken further to 2.0 MPa, wide cavities representing hemorrhage can be detected (in a healthy brain) (95). These extravasations can range from minor to even serious, which depends on the MBs concentration (8). Moreover, neuronal damage (dark appearance) is also possible when optimal parameters are exceeded (8). Therefore, choosing the appropriate optimal parameters and MBs dosage is critical to ensure safe BBB/BTB disruption with meaningful results (8). **Figure 3** highlights some of these possible side-effects both at low and high pressure.

### Tumor Control and Survival in Glioblastoma Models

The number of sessions that LIFU+MBs is applied has no effect on survival times. The latter is similar for both a single or double course (53) without increasing mortality and morbidity (54). When it is combined with a therapeutic drug, it results in an increased ability to inhibit tumor growth and size and increase survival in glioblastoma models (summarized in **Table 6**). This can be due to the increased availability of therapeutics in the targeted tumor volume which translates into increased apoptosis rates, leading to an increase in tumor cell damage (67, 70, 73). Ki-67 positive cells were seen to dramatically decrease following the administration of Dox (54, 55), PPNP (70), and PTX-liposome (73) after LIFU+MBs mediated BBB/BTB disruption, indicating significant inhibition of glioblastoma proliferation. Together with etoposide, LIFU+MBs resulted in 45% reduction in tumor growth, increasing survival up to 25 days (30% increase) compared to 19 days in other groups including etoposide alone or LIFU alone (54). Similar results (40% increase in survival) were observed after the delivery of liposomes containing PTX and anti-PD-1 antibody, which initiated ROS generation by ultrasound irradiation at glioblastoma sites (94). Delivery of AMPTL (NPs containing ROS and PTX with an autophagy inhibitor modified with angiopep-2 peptide) with LIFU+MBs showed significant inhibition of tumor growth and extended survival from 28 days to 50 days compared with controls with the slowest weight loss (38). After the delivery of Cabazitaxel with LIFU+MBs, tumor growth was significantly slowed, where its size was about one-third compared to the controls after 3 weeks (65). In the same way, when treated with Herpes Simplex Virus type 1 Thymidine Kinase/ganciclovir VCMBs (VEGFR2-targeted cationic MBs) in combination with LIFU, tumors were relatively smaller after 25 days with a prolonged survival of 28 days compared to 20 days in controls (72). When drugs which have poor water solubility were delivered in combination with LIFU+MBs, for instance, liposomes containing PTX, there were more apoptotic cells with a slow increase in tumor volumes (73). The delivery of DVDMS (sinoporphyrin sodium) with LIFU mediated BBB/BTB disruption showed delayed tumor growth with significant decrease in PCNA-positive cells levels and a maximum survival time extension to 39 days compared to 26 days in controls (64). Delivery of PTX liposomes increased median survival time up to 46.5 days (20.8% increase) compared to controls, while extending maximum survival up to 58 days (34.9% increase) (73). When ABX (PTX) was tested in 2 mice models with 2 different glioblastoma cell-lines, the mice that received LIFU+MBs treatment exhibited an improved survival of nearly about double median survival time (35 days) compared to controls (20 days) in the MES83 cell line. However, this was not the case when using GBM12 cell line, probably because ABX has a decreased sensitivity to this particular cell-line (58). Delivery of Car with LIFU+MBs resulted in relatively smaller tumor size with a 50% and 25% longer survival as compared to controls and Car only in U87 and PDX models, respectively, where PDX models are usually considered more aggressive (63). The same combination drastically increased the glioblastoma doubling time and survival up to 66% and 48%, respectively, compared to controls and Car only (47). This delay in tumor growth was also seen when shRNA-loaded liposomes were delivered to glioblastoma-bearing rats, where the tumor volume was about 10 times greater than the LIFU+MBs group (67). Remarkable inhibition of glioblastoma growth (91.1%) was seen when Dox was delivered with hollow MSN NPs (HCu) and LIFU+MBs, with more tumor cell damage and apoptosis compared to controls (69). This increased the median survival time up to 52 days compared to 24 days, 32 days, 42 days, and 35 days in LIFU alone, Dox alone, Dox+LIFU, and Dox-HCu groups, respectively (69). The median survival of mice treated with PPNP (PTX NPs) with LIFU+MBs group increased to 37 days compared with 26 days for the control group; indicating systemic administration of PPNP with LIFU+MBs could remarkably improve glioblastoma survival rate and prolong the total survival time with more apoptotic tumor cells (70). As discussed earlier, different acoustic pressure can have different outcomes. Similarly, when a higher pressure is used, it can result in a better glioblastoma growth inhibition and a significantly better animal survival which have been demonstrated with the delivery of brain penetrating nanoparticles (BPN) where there was a 15% improvement when 0.80 MPa was used instead of 0.60 MPa and 64% improvement compared to BPN alone in F98 models (26). This study also showed that the higher pressure resulted in smaller tumor volumes with more defined borders compared to the relatively lower pressure which had diffused borders after treatment (26). This particular study showed no difference in survival between 0.60 MPa (the lower pressure in this study) and BPN alone (26).

### Inflammation and Immune Response

There is a lot of evidence to support an inflammatory response following LIFU+MBs mediated BBB/BTB disruption (19, 43, 96–98) without evidence of vascular damage on histologic examinations (61). These responses are dependent on cavitation dose (99). It involves the elevation of pro-inflammatory cytokine levels together with microglial and astrocyte activation (61, 100–102). Microglial activation was confirmed *via* elevation of calcium-binding adaptor molecule 1 (Iba1) while astrocytic activation *via* increased glial fibrillary acidic protein (GFAP) (43, 61). Although the inflammation is immediate (91) and even with repeated sessions (102), it is usually mild (19) and resolves quite quickly with no such observations in the long term (47, 97, 99, 102). This inflammatory response could have happened due to exposure of blood constituents to the temporary BBB/BTB disruption (19), which is mediated through NF-κB pathways (101, 103), increasing the inflammatory markers such as chemotactic factors, heat-shock protein 70 (HSP70), and several pro-inflammatory cytokines namely TNFα, IL1a, IL1b, IL18, and IFNγ (8, 43, 91). A possible explanation of these factors is vasoconstriction which happens following MB mediated disruption leading to a slow perfusion (104). In addition to this, another pathway such as Akt signaling was also activated (43). These responses led to macrophage infiltration, which also resolved in several weeks (8, 91). With optimal parameters, and a lower dose of MB to avoid inertial cavitation, the severity of this inflammatory response can be controlled and lowered (8, 91, 103).

The brain, which was considered to lack immunity since the early 20th century, has now contradictory evidence to prove the trigger of the innate and cellular immune response following sonication (15). As discussed above, the transition of CD68+ macrophages from the bloodstream to the brain parenchyma (8, 91), is an indication of innate immune response. On the other hand, an increase in T cell population, CD3+ CD8+ lymphocytes infiltration and cytotoxic T-lymphocyte/Treg ratio in C6 glioblastoma after LIFU+MBs is an indicator of the cellular immune response (8, 85, 91). Infiltration of CD3+ CD8+, CD3+ CD4+, and CD4+ CD25+ lymphocytes were also noted after IL-12 injection in combination with LIFU+MBs (8, 85, 91). These immunological reactions were limited to the brain without any change in systemic distributions (8, 85). Before considering these effects, it is important to note that C6 glioma is not similar to the nature of human gliomas, where immune reactions are common in the former compared to the latter (91). In a rat preclinical model, CD4+ and CD8+ lymphocytes were seen to significantly increase after 7 days when 0.81 MI was used (56). This study did not observe any change in CD68+ macrophage or FOXP3+ lymphocyte counts. On the contrary, when they were exposed to 0.61 MI, there were no significant increases in these lymphocytes/macrophages after 7 days (56). It is deduced that higher LIFU+MBs exposure level potentially can trigger TIL (tumor infiltrating lymphocytes)-related immune response (56). **Figure 3D** shows some of the infiltration of macrophage and T cells at higher acoustic pressures.

## LIFU Mediated Liquid Biopsy

The BBB/BTB hinders the influx of substances into the CNS while simultaneously hindering the egress of tumor biomarkers into the peripheral circulation (105). This poses a major limitation in the molecular diagnosis of glioblastomas leaving only stereotactic biopsies as a way for analysis which is both invasive and poses the risk of infiltration to other parts of the brain, without forgetting the risk of infection (105). Fortunately, in the same way that BBB/BTB disruption allows substances to cross through the BBB/BTB into the brain, there is also the possibility of other substances to spill in the intravascular circulation vice-versa (as shown in **Figure 3C** with orange full arrow). This was also seen when the hydrophobic drug Cabazitaxel refluxed back in the bloodstream after BBB/BTB disruption (65). This bi-directional movement was termed as a “two-way transfer” where the application of LIFU+MBs allowed the detection of brain tumor biomarkers which is generally difficult to obtain (85, 92). This liquid biopsy (LB) resulted in a significant increase in plasma green fluorescent protein (eGFP) mRNA level (106) as well as glial fibrillary acidic protein and myelin basic protein in the peripheral circulation (92) both in glioblastoma and normal brain, respectively. The same technique showed increased concentration of cell-free DNA, neuron-derived extracellular vesicles, and brain-specific protein S100b when it was applied in clinical trials for glioblastoma (107). These results suggest a very important place of LB to be considered while precisely diagnosing glioblastomas and its different subtypes in the future.

## LIFU in the Clinic for Glioblastoma

In the early days, delivering FUS to the brain required the removal of the skull which limited this procedure to be performed solely in the operating room (19). The multidisciplinary technique of coupling MRI to LIFU (magnetic resonance guided FUS; MRgFUS), together with the use of transcranial FUS to achieve greater precision while targeting areas in the brain has lifted those obstacles in the current practice (18, 108). MRgFUS is mainly used in clinical applications for brain treatment (18). Its usage has already been approved by the FDA for essential tremor and tremor dominant Parkinson’s disease in 2016 and 2018, respectively (18, 19). Following that big achievement, and after being in the pre-clinical development for more than 20 years, LIFU+MBs mediated BBB/BTB disruption has just entered numerous clinical trials for glioblastomas (8, 31, 34, 85, 91, 109).

Currently, there are three devices including EXAblate Neuro 4000 220 kHz (InSightec, Haifa, Israel), NaviFUS (NaviFUS Taipei, Taiwan), and SonoCloud-9 (CarThera, Paris, France) which utilizes either implantable US devices or extracranially applied FUS devices to open the BBB/BTB with millimeter precisions (19, 91). The transducer is fixed on the scalp of the patient *via* a stereotactic frame and the space in between is eliminated using degassed water to minimize ultrasound attenuation (18). Pre-treatment MR images are loaded into the MRgFUS to identify the targeted region in real time (18). Furthermore, real time MR thermometry can be used to detect and control tissue temperatures (18, 108). After the opening of the BBB/BTB, it can be confirmed with gadolinium-enhanced CE-MRI and therapeutics can be delivered in the time window while the BBB/BTB is still open (18, 34, 56).

The use of LIFU mediated BBB/BTB disruption has been exploited in the clinic, which was well-tolerated even after the delivery of Car, TMZ, Dox, and fluorescein (56, 110–113). Similar to the preclinical results, the degree of BBB/BTB disruption increased with acoustic pressure (112). Patients had no clinical/radiologic adverse effects or any neurotoxicity (56, 111–113). The disruption resulted in 15-20% increase in contrast enhancement almost instantaneously and resolved after about 20 to 24 h (56, 111). In 6 patients following glioblastoma surgery, LIFU+MBs was applied within a 2-cm margin (110), a frequent area for tumor recurrence due to infiltrative cells presence and intact BBB (8). None of them had any adverse effects for 6 cycles showing that BBB/BTB disruption can be achieved safely and repeatedly (110). This study also suggests using both T1 weighted imaging and T2 weighted imaging to confirm BBB/BTB disruption which increases the accuracy up to 92.4% (110). When Car was delivered, progression-free survival and median overall survival were increased in patients who displayed BBB/BTB disruption (112). When compared to intra-operative fluorescence, it showed a positive correlation with results obtained post-sonication (113). LIFU+MBs were applied with exposures of 0.48, 0.58, and 0.68 MI in 6 patients with recurrent glioblastoma with treatment sessions of 95 min on average (56). There were no adverse effects related to the procedure and no immunological response observed in these patients (56). New development has recently led to the clinical trial of an implantable device which can predict the grade of BBB/BTB disruption with the aid of an algorithm (114) and the use of neuro-navigation (56). Clinical experience with repeated application of LIFU+MB is limited (34), and future research is needed to obtain significant conclusions. **Table 7** listed the few clinical trials of LIFU+MBs mediated BBB/BTB disruption in glioblastoma patients in the last 5 years.

**Table 7:** Clinical trials exploiting LIFU+MBs.

Sample Size	Description	Ref
6 patients with rGBM	Dose-escalating pilot trial using a device combining neuronavigation and a manually operated frameless FUS system to treat rGBM patients Outcomes: Safe and tolerable for all patients in the study. BBB at the target regions were opened successfully. Higher BBB permeability with higher energy of LIFU. No immunological response 7 days after procedure.	(56)
6 patients with resected GBM	LIFU applied within 2 cm margin in 145 BBBD trials (various brain locations) following T1 (90.3%) and T2 (64.1%) weighted GRE/MRI Outcomes: Well-tolerated. Repetitive procedure at the same target showed to be accurate and safe with 92.4% BBB disruption when T1 and T2 were combined.	(110)
4 patients with infiltrating gliomas	LIFU was applied in 9 to 31 subspots with increasing acoustic energy (3.38 W to 24.55 W) followed by fluorescein injection Outcomes: Well-tolerated. Safe, localized, and controllable BBB opening. Increase in fluorescein accumulation upon the use of LIFU+MBs.	(113)
5 patients with high grade glioma	LIFU followed by administration of liposomal doxorubicin and temozolomide prior to resection and quantification of drug in resected tissue samples. Outcomes: Safe and feasible with no clinical or radiologic procedure related side effects immediately or on 3 months follow up. Immediate 15-20% increase in contrast enhancement on T1 with resolution up to 20 h later.	(111)
19 patients with rGBM	LIFU followed by Carboplatin administration every 4 weeks until dose-limiting toxicity, severe adverse event, or disease progression evidence observed. Outcomes: Well-tolerated procedure with no drug related toxicity. Patients with successful BBB disruption showed increase in progression-free survival and median overall survival. The degree of BBB/BTB disruption increased with increasing acoustic pressure.	(112)
15 patients with rGBM	Application of LIFU for 40 ultrasound treatments up to 6 times (0.5 MPa – 1.1 MPa) with a dose increment of 0.15 MPa unless evidence of tumor progression was observed Outcomes: The main aim was to assess an algorithm with an implantable device to predict BBB opening grade (Grade 0, 1, 2, and 3). It predicted opening in gray matter with a probability of 3.33 times higher than white matter. The results showed a 10% chance of opening the BBB with a pressure <0.15 MPa compared to a 31.70% chance with a pressure >0.6 MPa.	(114)
9 patients with GBM	Collection of blood samples in these patients following MRgFUS mediated BBB/BTB disruption and patients with Alzheimer’s disease as control group for liquid biopsy. Outcomes: This technique enhances the signal for circulating brain-derived biomarkers (plasma cfDNA, neuron-derived extracellular vesicles, and brain-specific protein S100b). cfDNA-mutant copies of isocitrate dehydrogenase 1 (IDH-1) were increased.	(107)

GBM, glioblastoma multiforme; rGBM, recurrent GBM; GRE/MRI, gradient echo MRI; cfDNA, circulating free DNA.

## Concluding Remarks and Future Perspectives

One of the biggest challenges that we still face in this current era is the struggle for the successful delivery of anti-cancer therapeutics across the BBB/BTB to effectively treat glioblastomas. The latter protects cancerous cells from systemically administered therapeutics making pharmaceutical research problematic (9). With a considerably brief history, LIFU+MBs mediated BBB/BTB disruption has evolved significantly (**Box 5**), attracting much attention in recent years through its ability to improve therapeutics delivery and uniform distribution in these lesions thereby improving tumor control and survival. This acute interest has demonstrated several promising results with existing therapeutics and new innovating ones where it evolved from solo application to a multi-disciplinary approach incorporating real time monitoring, neuro-navigation, MRgFUS, and other tools (**Box 6**). As we seek to better understand this modality of delivery, innovative studies are required especially with those drugs with potential high toxicity against glioblastomas while simultaneously being unable to cross the BBB/BTB. Generally, the results that are obtained *in vitro* are not often reflected *in vivo*. Therefore, this novel technique opens the door to all those therapeutics that were previously considered not feasible or safe for further in-depth investigations. The different established existing methods such as paracellular, transcellular, transport proteins, efflux proteins, receptor-mediated transcytosis, adsorptive transcytosis, and cell mediated transcytosis pathways (115, 116) can be combined with LIFU+MBs mediated BBB/BTB disruption to facilitate the delivery of therapeutics to increase the rate of success in glioblastoma treatment. Moreover, specific ligands to target specific pathologic cell markers can be employed for a greater accuracy of targeting in combination with LIFU+MBs mediated BBB/BTB disruption for synergistic results.

Box 5 **Strategies exploited by LIFU+MBs mediated BBB/BTB disruption.**

- Drug delivery (where it has shown tumor control and increased survival)
- Liquid biopsies (where it can help to pre-establish molecular profiling of specific glioblastomas for tailor-made treatment strategies)

Box 6 **Multi-disciplinary innovations accompanying LIFU+MBs mediated BBB/BTB disruption.**

- MRgFUS
- MR thermometry
- Neuro-navigation
- Real time MBs activity monitoring (passive cavitation detection)
- MBs evolution (conjugation of drugs to nanoscale bubbles)
- *In vivo* imaging
- Ablation of MBs to produce reactive oxygen species

Of particular note, concerns relating to safety are aspects that should be thoroughly investigated. With that said, when opening the BBB/BTB, special attention should be given to the dosage of therapeutics, as there is a possibility of considerable amount of drug accumulation giving rise to adverse effects (**Box 7**). Therefore, sub-therapeutic doses can be first determined (54), to avoid circumstances that can lead to drug-related toxicity. Second, pre-investigations to fine-tune BBB/BTB disruption parameters should also be considered. This will help optimize acoustic pressures and MB dosage, confining the latter to the blood vessels and avoiding undesired inertial cavitation. This will minimize common side-effects, in particular, erythrocyte extravasations (85). Investigations with parameters close to tissue damage limits but without exceeding the limit can yield the best efficacy in drug delivery. This will be beneficial to induce an appropriate anti-cancer immune response while maintaining a safe and effective treatment at the same time (56). To sum up, LIFU+MBs mediated BBB/BTB disruption is an excellent technique for drug-delivery in glioblastomas and its surrounding infiltrative regions, where the effects are immediate while being safe at the same time. It offers both higher concentration and minimal side effects, which most pharmacologic researchers aim for. In addition to its feasibility in liquid biopsies of glioblastomas, new innovations and combinations with this technique remain to be seen.

Box 7 **Possible adverse effects of LIFU+MBs mediated BBB/BTB disruption.**

- Microhemorrhage or macrohemorrhage (RBC extravasation)
- Inflammatory response
- Immune cells infiltration
- Damaged neurons
- Toxicity (due to excess drug delivery)

## Author Contributions

RM wrote the original draft. RZ and YT supervised the project. BW, JZ, and XC contributed to major revisions to the manuscript. All authors contributed to the article and approved the submitted version.

## Funding

This work was supported by National S&T Major Project (2018ZX10301201) and NSFC (82027803).

## Conflict of Interest

The authors declare that the research was conducted in the absence of any commercial or financial relationships that could be construed as a potential conflict of interest.

## Publisher’s Note

All claims expressed in this article are solely those of the authors and do not necessarily represent those of their affiliated organizations, or those of the publisher, the editors and the reviewers. Any product that may be evaluated in this article, or claim that may be made by its manufacturer, is not guaranteed or endorsed by the publisher.

## Abbreviations

ABC, ATP-binding cassette; AJs, adherens junctions; BBB, blood-brain barrier; BTB, blood-tumor barrier; Car, carboplatin; CI, cavitation index; Dox, doxorubicin; EB, Evans blue; ECs, endothelial cells; ECM, extracellular matrix; FUS, focused ultrasound; HIFU, high-intensity focused ultrasound; JAM, junctional adhesion molecule; LIFU, low-intensity focused ultrasound; LC-MS/MS, liquid chromatography-tandem mass spectrometry; MBs, microbubbles; MI, mechanical index; MRgFUS, magnetic resonance guided focused ultrasound; MW, molecular weight; NVU, neurovascular unit; PTX, paclitaxel; RBC, red blood cells; TJs, tight junctions; TMZ, temozolomide; ZO, zona occludens, US, Ultrasound.
